# The Effectiveness of Sequentially Delivered Web-Based Interventions on Promoting Physical Activity and Fruit-Vegetable Consumption Among Chinese College Students: Mixed Methods Study

**DOI:** 10.2196/30566

**Published:** 2022-01-26

**Authors:** Yanping Duan, Wei Liang, Yanping Wang, Sonia Lippke, Zhihua Lin, Borui Shang, Julien Steven Baker

**Affiliations:** 1 Department of Sport, Physical Education and Health Faculty of Social Sciences Hong Kong Baptist University Hong Kong China (Hong Kong); 2 Center for Health and Exercise Science Research Hong Kong Baptist University Hong Kong China (Hong Kong); 3 The National Physical Fitness Lab Hubei Institute of Sport Science Wuhan China; 4 Department of Psychology & Methods Jacobs University Bremen Bremen Germany; 5 Sport Section Wuhan University Wuhan China; 6 Department of Social Sciences Hebei Sport University Shijiazhuang China

**Keywords:** web-based intervention, physical activity, fruit-vegetable consumption, college students, health action process approach, mixed methods, quantitative research, qualitative research

## Abstract

**Background:**

Web-based interventions for multiple health behavior change (MHBC) appear to be a promising approach to change unhealthy habits. Limited research has tested this assumption in promoting physical activity (PA) and fruit-vegetable consumption (FVC) among Chinese college students. Moreover, the timing of MHBC intervention delivery and the order of components need to be addressed.

**Objective:**

This study aims to examine the effectiveness of 2 sequentially delivered 8-week web-based interventions on physical activity, FVC, and health-related outcomes (BMI, depression, and quality of life) and the differences in the intervention effects between the 2 sequential delivery patterns. The study also aims to explore participants’ experiences of participating in the health program.

**Methods:**

We conducted a randomized controlled trial, in which 552 eligible college students (mean 19.99, SD 1.04 years, 322/552, 58.3% female) were randomly assigned to 1 of 3 groups: PA-first group (4 weeks of PA followed by 4 weeks of FVC intervention), FVC-first group (4 weeks of FVC followed by 4 weeks of PA intervention), and a control group (8 weeks of placebo treatment unrelated to PA and FVC). The treatment content of two intervention groups was designed based on the Health Action Process Approach (HAPA) framework. A total of four web-based assessments were conducted: at baseline (T1, n=565), after 4 weeks (T2, after the first behavior intervention, n=486), after 8 weeks (T3, after the second behavior intervention, n=420), and after 12 weeks (T4, 1-month postintervention follow-up, n=348). In addition, after the completion of the entire 8-week intervention, 18 participants (mean 19.56, SD 1.04 years, 10/18, 56% female) who completed the whole program were immediately invited to attend one-to-one and face-to-face semistructured interviews. The entire study was conducted during the fall semester of 2017.

**Results:**

The quantitative data supported superior effects on physical activity, FVC, and BMI in the 2 sequential intervention groups compared with the control group. There were no significant differences in physical activity, FVC, and health-related outcomes between the 2 intervention groups after 8 weeks. The FVC-first group contributed to more maintenance of FVC compared with the PA-first group after 12 weeks. Four major themes with several subthemes were identified in the qualitative thematic analysis: PA and FVC behavior, health-related outcomes, correlates of behavior change, and contamination detection.

**Conclusions:**

This study provides empirical evidence for the effectiveness of sequentially delivered, web-based MHBC interventions on PA and FVC among Chinese college students. The timing issue of MHBC intervention delivery was preliminarily addressed. Qualitative findings provide an in-depth understanding and supplement the quantitative findings. Overall, this study may contribute considerably to future web-based MHBC interventions.

**Trial Registration:**

ClinicalTrials.gov NCT03627949; https://clinicaltrials.gov/ct2/show/NCT03627949

**International Registered Report Identifier (IRRID):**

RR2-10.1186/s12889-019-7438-1

## Introduction

### Background

Considerable evidence indicates a high prevalence of physical inactivity and insufficient consumption of fruit and vegetables among college students [1]. In China, especially, recent studies have revealed that about 40% of Chinese college students do not meet the 150 accumulated minutes of moderate physical activity (PA) per week recommended by the World Health Organization [2,3], and more than half of this population does not consume the recommended 5 servings (400 g) of fruit and vegetables per day [4,5].

College students, who are in a crucial transition stage from late adolescence to adulthood, adopting such unhealthy behaviors can increase the risk of many chronic diseases (eg, cardiovascular diseases, obesity, or type 2 diabetes) and jeopardize their mental health (eg, increase the risk of depression) [6,7]. Therefore, promoting PA and fruit-vegetable consumption (FVC) in this population is essential. Over the past 2 decades, much evidence has shown that multiple health behavior change (MHBC) interventions can promote both PA and healthy diet among college students [1,8,9]. Thus, MHBC interventions for college students are promising for supporting long-lasting behavior changes into late adulthood.

With the burgeoning use of the internet, web-based MHBC intervention programs have been widely applied in various populations [10-12]. Compared with traditional face-to-face, analogous-delivered modes, using the internet to deliver MHBC interventions has been demonstrated to have a series of advantages, such as accessibility, scalability, cost-effectiveness, and convenience [13]. For college students, who form the majority of the internet users, the acceptability and effectiveness of web-based MHBC interventions for promoting PA and FVC have been proven by a growing body of evidence [1,9,14]. However, most of the existing studies have been conducted in Western countries, while there is limited research on Chinese college students.

One debatable question in MHBC research is how to deliver MHBC interventions (ie, delivery timing) to achieve more robust treatment effects and whether the order of the sequential intervention contents makes a difference [15]. Opinions differ, and the evidence is limited and inconsistent to date. One view suggests that multiple health behaviors typically coexist as behavioral clusters or bundles [16-19]. For example, one risk behavior (eg, sedentary behavior) often occurs with other risk behaviors (eg, excessive intake of fat and sugar, smoking, or alcohol addiction) or one health-protective behavior (eg, physical activity) coexists with other health-protective behaviors (eg, FVC). This interconnection of health behaviors can generate synergistic or additive effects, contributing to the reinforcement of the treatment effects when changing them simultaneously [16-18]. Furthermore, this approach is shorter and less costly [18]. In contrast, some researchers argue that simultaneous intervention delivery may be overburdened, as it requires individuals to make considerable efforts to self-regulate to adopt multiple health behaviors [20,21]. In addition, a simultaneous approach may not address any particular behavior in sufficient depth, decreasing the potential effects of the intervention. Therefore, sequential delivery may be more suitable [19,20]. This design often requires delivering interventions over a longer period, which potentially increases costs, and if the design lacks motivation strategies, participation and adherence will suffer [22]. In addition to these viewpoints, a review of 6 studies suggested that both simultaneous and sequential approaches can be considered equally effective in MHBC interventions [23]; thus, the main question relates more to the sequential order of the intervention components.

However, previous MHBC studies focused mostly on the combination of heterogeneous categories of behaviors (eg, risk behaviors plus health protecting behaviors). When targeting PA and FVC, which are both health-protecting behaviors, some evidence has indicated that there is a gateway or carry-over effect between PA and FVC [21,24,25]. In a sequential design, changing the first behavior (PA/FVC) may result in increased self-efficacy or self-regulation to undertake the second behavior (FVC/PA), thereby contributing to successful changes in both behaviors [21,24,25]. In addition, using a sequential design may ensure that each behavior can be adequately addressed, and this is particularly important for college students who are at a critical life transition to establish or sustain favorable lifestyles in the long term [8]. Given these findings, it is possible that a sequential approach may be more effective for interventions that address PA and FVC behavior among college students. Nevertheless, the question related to 2 sequential delivery patterns (PA-first vs FVC-first), of which one would contribute more to improvements in health behaviors and health-related outcomes, is still unclear. Further examination of this aspect is warranted.

### This Study

The health action process approach (HAPA) model was used as the theoretical framework of intervention in this study [12,26,27]. The HAPA model postulates two distinctive stages (motivational and volitional) for the process of behavior change, where individuals may experience a dynamic process from generating a behavioral intention to performing and maintaining a specific health behavior [28]. A series of psychosocial factors are considered crucial in the behavioral change process. During the motivational stage, the primary task is to form a behavioral intention, where action self-efficacy, outcome expectancies, and risk perceptions are proposed as contributory antecedents of intention. Once individuals have initiated behavioral intention, volitional factors (action planning and coping planning, maintenance, and recovery self-efficacies) and external resources (social support) play imperative roles in bridging the intention-behavior gap to facilitate behavioral execution and maintenance. The applicability of the HAPA model in promoting various health behaviors among adults has been widely approved [16,29,30].

The mixed methods approach can combine the merits of both quantitative and qualitative methods (ie, integrating the power of stories and the power of numbers) and compensate for their respective limitations [31]. Therefore, this study uses a sequential mixed methods design, including a randomized controlled trial (RCT) and face-to-face interviews. In particular, the RCT aims to quantitatively examine the effectiveness of web-based interventions in promoting PA and FVC behavior and health-related outcomes among Chinese college students. We hypothesize that (1) both intervention groups (PA-first and FVC-first) would show more changes in PA (metabolic equivalent [MET]-min/week) and FVC (Portion/ day) compared with the control group; (2) both intervention groups would show more changes in health-related outcomes (BMI, depression, and perceived quality of life) compared with the control group; and (3) 2 intervention groups would differ significantly in PA (MET-min/week), FVC (portion/day), and health-related outcomes at 8 and 12 weeks. With regard to the one-on-one and face-to-face qualitative interviews, we aim to explore college students’ experiences and perceptions of participating in the web-based MHBC intervention program.

## Methods

### Quantitative Study: An RCT

#### Participants and Procedure

Considering the feasibility and limited resources, an RCT was adopted instead of a cluster RCT with a standard 3-arm, parallel, double-blinded design. The study participants were undergraduate students from a university in the central region of China. The sample size was calculated using G*Power software (Version 3.1). As this study aimed to improve MHBC, PA and FVC were treated as *coprimary outcomes*, and the family-wise error rate was not necessary to be controlled for the sample size calculation [32,33]. Considering the effect size of PA change was smaller than that of FVC in our previous studies, the sample size estimate in this study was based on an average effect size of 0.45 on PA only (Cohen *d*) [26,29] to ensure robust analyses for the treatment effects on both outcomes (ie, conjunctive power) [32,33]. Therefore, to provide a power of 0.8 (1-β) with an *α* of .05, 79 participants were required for each group. Assuming a dropout rate of 40% to the posttest (dropout rates ranged from approximately 30% to 50% in previous studies [26]), a total of 396 participants (132 per group) were needed.

A total of 634 college students were contacted in their first general physical education (PE) classes with the assistance of PE lecturers during the fall semester of 2017. In China, college students are required to take PE courses based on the national education guideline [34]. Therefore, it was feasible to recruit students from different departments via their PE classes. Once students expressed interest, they were provided with a hard copy of the study consent form and were invited to complete the web-based registration (including sociodemographic information) by scanning a quick response (QR) code. Among the 634 participants, 565 (89.1%) college students (aged ≥18 years) met the following eligibility criteria: (1) were not collegiate athletes or majoring in any sport-related subjects, (2) were not vegetarians, (3) had no restrictions on physical mobility (eg, heart diseases, stroke, or disability) or FVC (eg, fruit allergies or diabetes), and (4) were able to use a computer or laptop and mobile phone and had access to the internet.

After qualification screening, all 565 eligible students were randomly allocated to 1 of 3 groups. The random number list was generated at the backend management system of the website platform. The three groups consisted of intervention group 1 (PA-first group: first 4-week intervention addressing physical activity, followed by a 4-week intervention addressing FVC; 189/565, 33.5%), intervention group 2 (FVC-first group: first 4-week intervention addressing FVC, followed by a 4-week intervention addressing physical activity; 198/565, 35.0%), and a placebo control group (8-week placebo treatments, which were not relevant to changing actual PA and FVC behavior; 178/565, 31.5%). One week after randomization, participants were invited to attend the session once a week for approximately 25 minutes each time for 8 weeks. All participants were asked to complete the weekly health sessions independently and not to discuss the content of the health sessions with their classmates, roommates, and friends.

**Figure 1:**
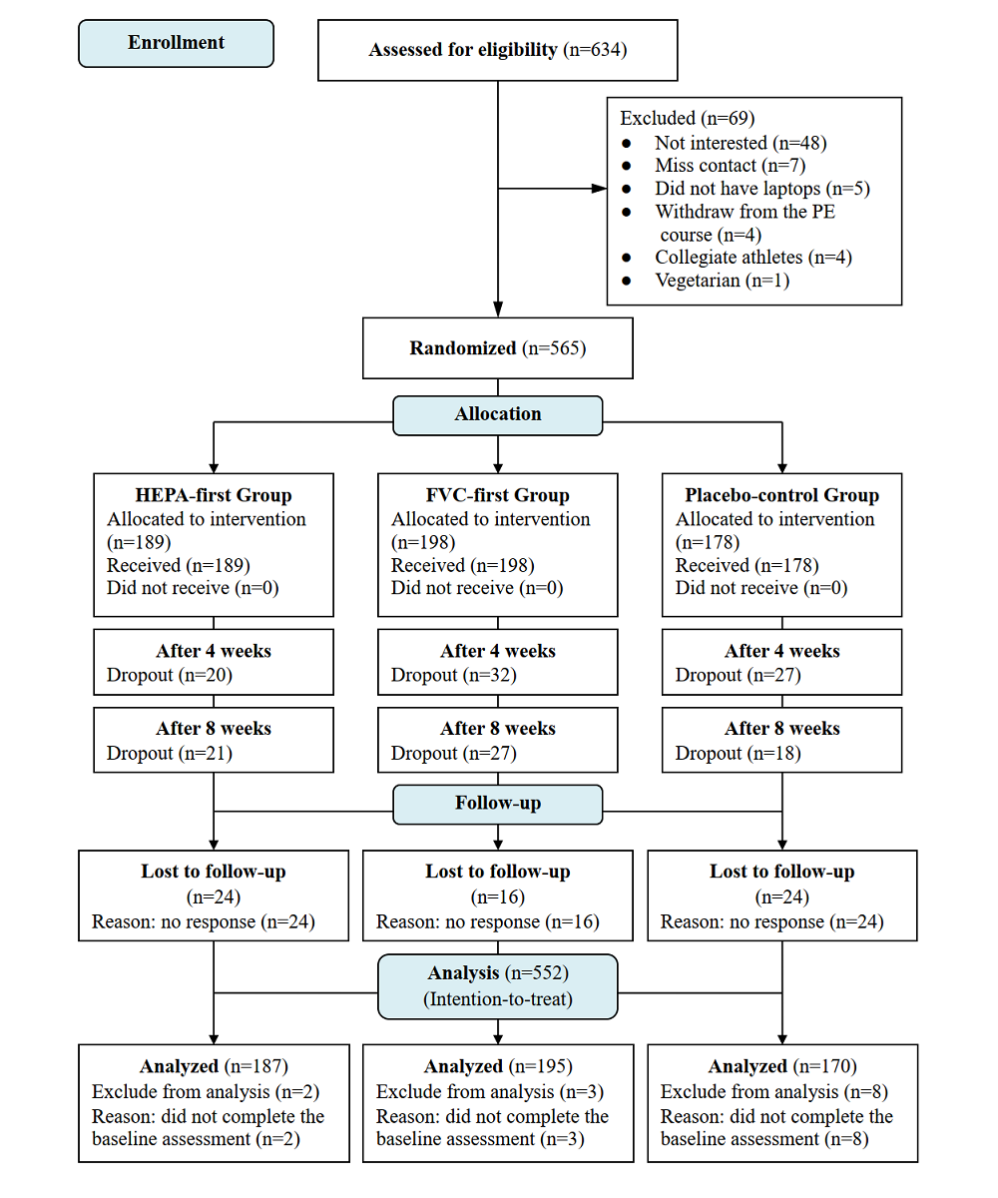
CONSORT (Consolidated Standards of Reporting Trials) flow diagram. FVC: fruit-vegetable consumption; PA: physical activity; PE: physical education.

In addition to attending the intervention session, all participants were asked to complete electronic questionnaires at 4 time points: baseline (T1, at the beginning of the intervention), after 4 weeks (T2, after the completion of the 4-week intervention on the first behavior), after 8 weeks (T3, after the completion of the 4-week intervention on the second behavior), and after 12 weeks (T4, 1-month follow-up after intervention completion; Multimedia Appendix 1). After excluding participants who did not complete the baseline assessment, the final sample consisted of 552 participants. Participant flow and retention are shown in Figure 1.

We obtained ethical approval from the research ethics committee of Hong Kong Baptist University (FRG2/15-16/032) and registered the study on ClinicalTrials.gov (NCT03627949).

#### Intervention

The intervention was designed based on the health action process approach (HAPA) model, which lasted for 8 weeks [9,12,27,28]. The PA-first module was designed in a previous project conducted by our research team [26,29]. In particular, the intervention content of the first 4-week period targeted the following HAPA-based constructs for physical activity: week 1 included risk perception, outcome expectancies, and goal settings (contributing to the formation and enhancement of PA intention); week 2 included development of action planning; week 3 included revision and adjustment of previous action planning and development of coping planning; and week 4 included revision and adjustment of previous coping planning and development of perceived social support. The same intervention constructs were then implemented to target the HAPA-based social cognitive determinants of FVC change in the second 4-week period. The earlier project only addressed one sequence of intervention delivery (PA-first) among Chinese college students [24]. This study extended the intervention delivery modules by adding an FVC-first module. Multimedia Appendix 2 shows the intervention variables and content for the 2 intervention groups and a placebo control group. Furthermore, a series of behavior change techniques were used in the weekly intervention sessions to facilitate the implementation and maintenance of PA and FVC [35] (Multimedia Appendix 3).

In addition, given the high dropout rate (31.6% for posttest and 71.2% for the follow-up test) in our previous study [24], several new strategies and approaches were implemented in this study to increase the attractiveness of the health program and maximize participant retention [36], including (1) restructuring the website page to match the preferences of young adults, for example, supplementing vivid pictures and redrawing the layout; (2) adding pop-up messages to prevent participants from missing a unit or a single item; and (3) using WeChat (a popular social media platform in China) to contact the participants and deliver the reminder messages. In addition, participants who adhered to the 8-week intervention and 4 time-point data collection would be provided with monetary incentive (US $8.0) as a reward.

#### Measures

##### Primary Outcomes: PA and FVC

PA was measured using the Chinese short version of the International Physical Activity Questionnaire (IPAQ-C) [37]. The IPAQ-C asked participants to report the frequency and duration of engaging in 3 intensities of PA in the past 7 days. The weekly MET-min of total PA were calculated using the following equation [37]:


*PA (MET-min/week) = 8.0 METs × vigorous intensity of PA (min/week) + 4.0METs × moderate intensity of PA (min/week) + 3.3METs × light intensity of PA (min/week)*

FVC was measured by using a 4-item scale, asking the participants to count the portions of fruit and vegetables they consumed on average during a typical day [38]. Each item (*raw vegetables*, *fruit*, *cooked or steamed vegetables*, and *fruit or vegetable juice*) had 11 options for the number of portions such as 0, 0.5, 1, 1.5, 2, and 2.5 until 5 or above. The total consumed portion was the sum of each item.

##### Secondary Outcomes: Health-Related Outcomes

BMI was measured by asking participants to self-report their body weight (kg) and height (m). BMI was calculated using the formula *BMI = weight (kg)/height squared (m^2^)* [39].

Depression was measured using the Chinese version of the Centre for Epidemiologic Studies Depression–10 Scale [40]. Participants were asked with the question stem as “In the past week how often I feel” followed by 10 items such as “...I was bothered by things that usually don’t bother me” (Cronbach *α*=.78). Answers were given on a visual analog scale (VAS) from rarely or none of the time (<1 day) *0* to most or all the time (5-7 days) *3* [40,41].

Perceived quality of life was assessed using the short version of the World Health Organization Quality of Life-BREF [42]. Respondents were first asked about their general quality of life as “How would you rate your quality of life?” with a VAS score ranging from (very poor=1) to (very good=5). The physical health subdomain with 7 items was also measured (Cronbach *α*=.71), such as “How satisfied are you with your ability to perform your daily living activities?” with a VAS score ranging from (very dissatisfied=1) to (very satisfied=5).

##### Demographic Information

Gender, age, relationship status (single or in a relationship), grade (freshman or sophomore or junior or senior), major, and self-reported health status (bad or medium or good) were included in demographic information.

All measurement instruments were translated into a Chinese version and validated in previous studies with Chinese college students [26]. Demographic information was collected only at registration (T0). All other measurements were measured at T1, T2, T3, and T4.

#### Statistical Analyses

The data were analyzed using SPSS (version 25.0; IBM Corporation). Analyses of variances, independent *t* tests, and chi-square tests were performed to examine whether the randomization was successful. Where there were significant differences across groups at baseline, the variables were treated as covariates in subsequent analyses. Data analyses for the intervention effects were performed using the intention-to-treat principle, with per-protocol analysis as a sensitivity test [43]. Missing values were addressed using the multiple imputation method with chained equations, except for dropouts, which were imputed with the baseline-observation-carried-forward approach [44]. A series of generalized linear mixed models were applied to evaluate the intervention effects on outcome measures at different time points, with time, group, and their interaction as fixed effects, and with individuals as random effects. On the basis of the −2 log likelihood and Akaike and Bayesian information criteria, an unstructured covariance structure was selected for the model estimation using a restricted maximum likelihood approach. For the post hoc comparison, considering that studies with co-primary outcomes may increase the type-II error rate and decrease the study power [33], the least significant difference method was used rather than other adjusted approaches (eg, Bonferroni) [45-47]. In addition, chi-square tests were used for post hoc comparisons. The 5% level (2-tailed) was used as the statistical significance cut-off point.

### Qualitative Study: Interviews

#### Study Design and Participants

On the basis of the guideline of descriptive phenomenology, a series of one-on-one and face-to-face semistructured interviews were conducted, which involved three types of questions (open-ended, closed-ended, and conformational) [48].

To reach theoretical saturation, based on the *rule of thumb* and *calculating the mean of selected qualitative studies*, 18 participants who completed the web-based MHBC interventions were randomly recruited from 2 intervention groups and 1 control group in the RCT (6 participants for each group).

#### Procedure and Data Collection

The research team jointly developed an interview guide (4 experts in the health psychology domain), including questions, prompts, and guides, based on suggestions from Bryman and Flick [48,49]. A detailed interview guide can be found elsewhere [50] (Multimedia Appendix 4).

One-on-one and face-to-face semistructured interviews were conducted after the completion of the 8-week web-based MHBC interventions. On the basis of the interview guide, the main question was used to invite the participants to talk freely, such as “What is your experience with the 8-week web-based health program?” Additional questions were asked during the conversation for clarification and elaboration, such as “If so, can you explain in more detail? What caused this change in your behavior?” Each interview was audio-recorded and lasted approximately 30 minutes. Each interviewee could obtain US $6.5 as a participation remuneration if they completed the interview.

#### Data Analysis

The audio-recorded interview data were transcribed orthographically and organized using NVivo (version 11; QSR International). Thematic analysis was used for data analysis, including six phases: familiarization with the data, generation of the initial codes, searching for themes, reviewing the potential themes, defining and naming themes, and producing the report [51]. Two members of the research team (WL and YW) independently conducted the first five steps (intercoder agreement=97%). All discrepancies in any aspect of the analysis process (eg, defining the potential themes and subthemes) were discussed by three members of the research team (WL, YW, and YD) until consensus was reached. Both inductive and deductive processes were involved in the thematic analysis process. Finally, to guarantee the credibility and trustworthiness of the qualitative study, the entire procedure followed a set of principles, including sensitivity, commitment, rigor, transparency, coherence impact, and importance (Multimedia Appendix 5) [52]. To guarantee that this study complied with qualitative reporting standards, the 32-item Consolidated Criteria for Reporting Qualitative Studies (COREQ) checklist was used [53] (Multimedia Appendix 6).

## Results

### Quantitative Study Results

#### Randomization Check and Sample Characteristics

A randomization check indicated that there were no significant differences in baseline characteristics across the 3 groups in relation to gender, age, study year, relationship status, and self-reported health status (*P*=.37-.83). Moreover, the 3 groups did not differ significantly in all continuous and categorical variables with regard to the physical activity, FVC, and health outcomes at baseline (*P*=.098-.93). Therefore, the randomization was successful.

A description of the study sample is provided in Table 1. A total of 552 valid respondents were recruited from 28 different departments (the total number of university departments is 34), including 322 (58.3%) females and 230 (41.7%) males, with the age ranging from 18 to 24 years (mean 19.99, SD 1.04 years). Most of the participants were freshmen and sophomores, that is 47.8% (264/552) and 41.5% (229/552) of the total sample, respectively. Among these participants, only 46 (8.3%) indicated being in a relationship. The average BMI was 20.41 kg/m^2^ (SD 2.45 kg/m^2^). Most participants (374/552, 67.8%) had a healthy weight, 23% (127/552) were underweight, and 9.2% (51/552) were overweight. In addition, 64.9% (358/552) of the participants indicated a medium level of self-reported health status.

**Table 1:** Sociodemographic information, PA^a^, FVC^b^, and health outcomes of the study sample at baseline.

Variable			Total (N=552)	PA-first (n=187)	FVC-first (n=195)	Control (n=170)
**Sociodemographic information**						
	Age (range 18-24 years), mean (SD)		19.99 (1.04)	20.07 (1.07)	19.96 (0.99)	19.93 (1.06)
	**Gender, n (%)**					
		Male	230 (41.7)	79 (42.2)	78 (40)	73 (42.9)
		Female	322 (58.3)	108 (57.8)	117 (60)	97 (57.1)
	**Grade, n (%)**					
		Freshman	264 (47.8)	86 (46.0)	90 (46.2)	88 (51.8)
		Sophomore	229 (41.5)	77 (41.2)	84 (43.1)	68 (40)
		Junior	46 (8.3)	18 (9.6)	16 (8.2)	12 (7.1)
		Senior	13 (2.4)	6 (3.2)	5 (2.6)	2 (1.2)
	**Marital status, n (%)**					
		Single	506 (91.7)	170 (90.9)	183 (93.8)	153 (90)
		In a relationship	46 (8.3)	17 (9.1)	12 (6.2)	17 (10)
	**Health status, n (%)**					
		Poor	17 (3)	5 (2.7)	9 (4.6)	3 (1.8)
		Medium	358 (64.9)	122 (65.2)	125 (64.1)	111 (65.3)
		Good	177 (32.1)	60 (32.1)	61 (31.3)	56 (32.9)
**PA** **and** **FVC** **, mean (SD)**						
	PA (MET^c^-min/week)		2124.23 (1244.42)	2180.22 (1314.89)	2074.45 (1191.03)	2119.73 (1229.34)
	FVC (Portion/day)		3.81 (1.75)	3.84 (1.70)	3.82 (1.87)	3.76 (1.68)
**Health-related outcomes**						
	BMI (range 15.62-32.88 kg/m^2^), mean (SD)		20.41 (2.45)	20.32 (2.34)	20.52 (2.62)	20.40 (2.39)
	Depression, mean (SD)		0.92 (0.69)	0.85 (0.63)	0.93 (0.73)	0.98 (0.72)
	Quality of life, mean (SD)		3.15 (0.67)	3.23 (0.63)	3.14 (0.67)	3.08 (0.71)

^a^PA: physical activity.

^b^FVC: fruit-vegetable consumption.

^c^MET: metabolic equivalent.

#### Intervention Effects on PA and FVC

**Figure 2:**
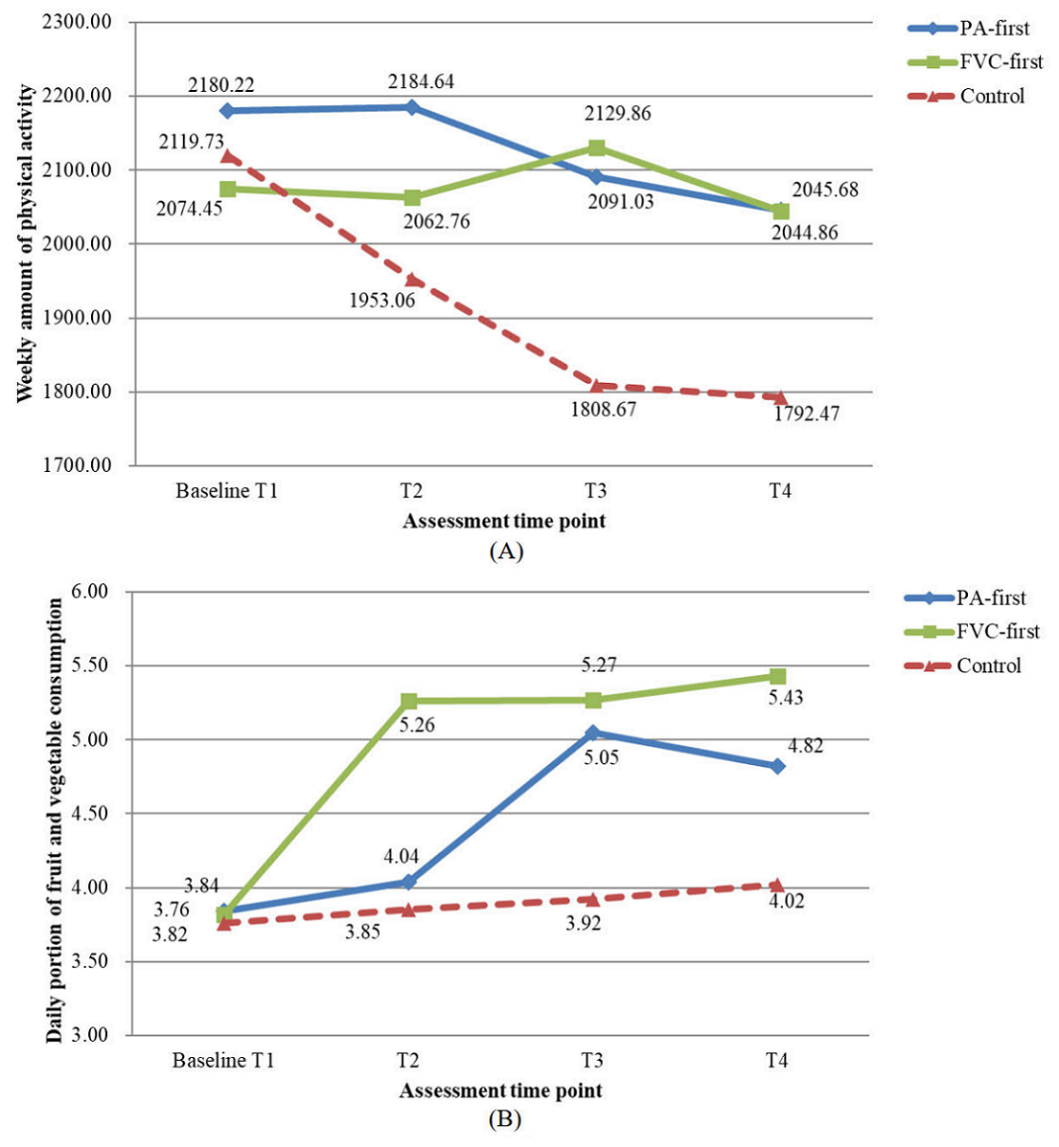
Mean values for 3 groups from timepoints T1 to T4. (A) weekly amount of physical activity (metabolic equivalent of task-min/week). (B) daily portion of fruit and vegetable consumption (portion/day). FVC: fruit-vegetable consumption; PA: physical activity.

Table 2 presents the results of the evaluation of the intervention effects on the weekly amount of PA and daily servings of FVC. Figure 2A and Figure 2B show the descriptive information of the 2 behaviors from T1 to T4. The results revealed that both health behaviors changed favorably and significantly over time (all *P*≤.001) and that there were significant differences in the time and treatment effects between the intervention and control groups in both PA (*P*=.02) and FVC (*P*<.001) behaviors. From the post hoc tests, we found small effect sizes for the intervention effects on PA (Cohen *d*=0.22-0.29) and small-to-medium effect sizes on FVC behavior (Cohen *d*=0.34-0.59).

To identify which intervention delivery schedule would be more effective in promoting health behavior change after 8 and 12 weeks, we compared the differences in each health behavior between the PA-first and FVC-first groups at T3 and T4. The results indicated that the 2 intervention groups did not differ significantly from each other in PA at either time point, but there was a significant difference in FVC between the 2 intervention groups (*P*=.014). The group receiving FVC instruction first had a significantly higher FVC after 12 weeks (T4).

**Table 2:** Results of the generalized linear mixed models with physical activity and fruit–vegetable consumption after 4, 8, and 12 weeks as outcome measures (n=552).

Time and group		PA^a^				FVC^b^		
		Values	*P* value	Effect size, Cohen *d*	Values		*P* value	Effect size, Cohen *d*
**Type III tests, *F*_549_**								
	Time×group	2.66	.015	N/A^c^	12.17		<.001	N/A
	Time	5.24	.001	N/A	36.40		<.001	N/A
	Group	2.05	.13	N/A	13.64		<.001	N/A
**After 4 weeks (T2)^d^, mean difference**								
	PA-first vs control	231.58	.041	0.22	0.19		.47	0.08
	FVC-first vs control	109.70	.33	0.10	1.42		<.001	0.58
	PA-first vs FVC-first	121.87	.26	0.11	−1.23		<.001	0.51
**After 8 weeks (T3)^d^, mean difference**								
	PA-first vs control	282.36	.018	0.25	1.13		<.001	0.44
	FVC-first vs control	321.19	.007	0.29	1.35		<.001	0.52
	PA-first vs FVC-first	−38.83	.74	0.03	−0.22		.41	0.08
**After 12 weeks (T4)^d^, mean difference**								
	PA-first vs control	253.21	.026	0.24	0.81		.02	0.34
	FVC-first vs control	252.39	.025	0.24	1.41		<.001	0.59
	PA-first vs FVC-first	0.83	.99	<0.01	−0.60		.014	0.25

^a^PA: physical activity (metabolic equivalent of task-min/week).

^b^FVC: fruit-vegetable consumption (portion/day).

^c^N/A: not applicable.

^d^Post hoc test: least significant difference; mean difference was significant at the .05 level.

#### Intervention Effects on Health-Related Outcomes

**Figure 3:**
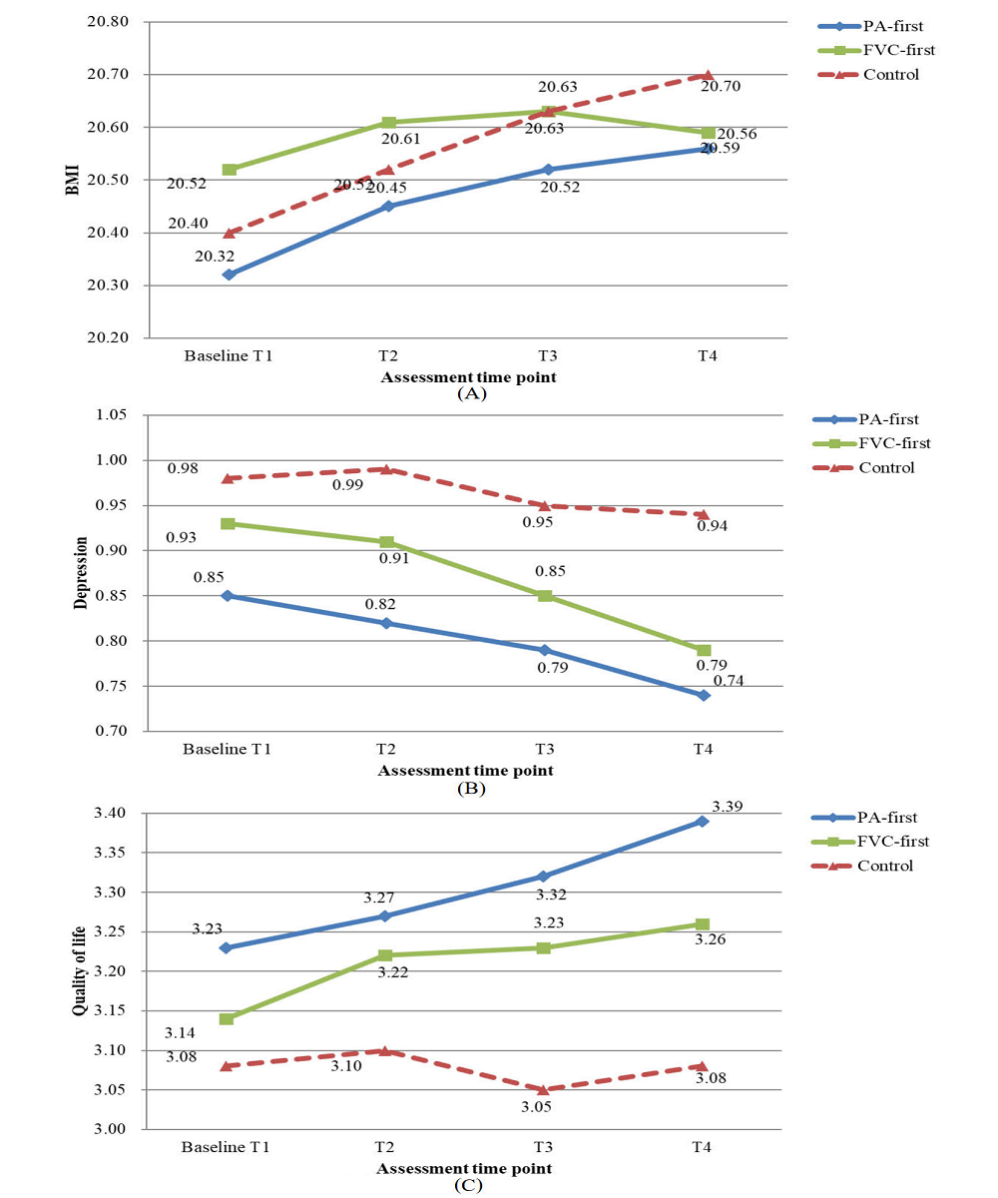
Mean values for 3 groups from timepoints T1 to T4. (A) BMI (kg/m2). (B) Depression. (C) Quality of life. FVC: fruit-vegetable consumption; PA: physical activity.

The descriptive results revealed that the intervention groups had a favorable time effect on health-related outcomes compared with the control condition (all *P*<.01; Table 3). For the time and treatment interaction, the difference was only significant for BMI (*P*=.03), and there were no significant differences between the intervention and control groups for depression (*P*=.60) and perceived quality of life (*P*=.07). From the post hoc tests, we found small effect sizes for the intervention effects on BMI (Cohen *d*=0.01-0.07), depression (Cohen *d*=0.07-0.31), and quality of life (Cohen *d*=0.07-0.47). Descriptive information is presented in Figure 3A-Figure 3C.

**Table 3:** Results of the generalized linear mixed models with health-related outcomes (ie, BMI, depression, and perceived quality of life) after 4, 8, and 12 weeks as outcome measures (n=552).

Time and group			BMI							Depression						Quality of life				
			Value		*P* value		Effect size, Cohen *d*		Value			*P* value		Effect size, Cohen *d*	Value			*P* value		Effect size, Cohen *d*
**Type III tests, *F*_549_**																				
	Time×group	2.34		.03		N/A^a^		.76			.60		N/A		1.95		.07		N/A	
	Time	18.29		<.001		N/A		8.21			<.001		N/A		6.69		<.001		N/A	
	Group	.15		.86		N/A		3.61			.03		N/A		6.69		.001		N/A	
**After 4 weeks (T2)^b^, mean difference**																				
	PA^c^-first vs control	−0.08		.76		0.03		−0.17			.01		0.27		0.16		.01		0.27	
	FVC^d^-first vs control	0.09		.71		0.04		−0.07			.28		0.11		0.12		.07		0.18	
	PA-first vs FVC-first	−0.17		.49		0.07		−0.10			.14		0.17		0.05		.46		0.07	
**After 8 weeks (T3)^b^, mean difference**																				
	PA-first vs control	−0.11		.64		0.05		−0.16			.02		0.25		0.27		<.001		0.42	
	FVC-first vs control	−0.02		.92		0.01		−0.10			.13		0.15		0.18		.009		0.26	
	PA-first vs FVC-first	−0.09		.70		0.04		−0.06			.37		0.10		0.09		.18		0.15	
**After 12 weeks (T4)^b^, mean difference**																				
	PA-first vs control	−0.14		.56		0.06		−0.19			.003		0.31		0.30		<.001		0.47	
	FVC-first vs control	−0.10		.66		0.04		−0.15			.02		0.23		0.18		.01		0.26	
	PA-first vs FVC-first	−0.04		.88		0.01		−0.05			.45		0.07		0.12		.07		0.19	

^a^N/A: not applicable.

^b^Post hoc test: least significant difference; mean difference is significant at the .05 level.

^c^PA: physical activity (metabolic equivalent of task-min/week).

^d^FVC: fruit-vegetable consumption (portion/day).

#### Dropout Analysis and Sensitivity Test

The dropout rate of participants was 14.3% (79/552) from T1 to T2, 14% (66/473) from T2 to T3, and 15.7% (64/407) from T3 to T4. The aggregated dropout percentage was 26.3% (145/552) from T1 to T3, and 37.9% (209/552) from T1 to T4. There were no between-group differences in the percentage of participants with incomplete data at T2, T3, and T4 (*χ*^2^_2_=1.3-3.4, *P*=.18-.51).

Overall, sensitivity analyses exhibited similar results for all dependent variables, except for the perceived quality of life, and the time and treatment effect was found to be statistically significant (*P*=.03) for both intervention groups compared with the control group (Multimedia Appendix 7). This indicated that our intervention significantly improved the perceived quality of life only for participants who adhered to the entire intervention. However, as noncompliance is inevitable in the real world, the results of the sensitivity test did not alter the findings of the primary analyses of the nonsignificant effect of time and treatment on the perceived quality of life (this was also consistent with the results of the likelihood-based estimation).

### Qualitative Study Results

#### Sample Characteristics

A total of 18 participants attended the interview study, including 10 females and 8 males, ranging in age from 18 to 22 years (mean 19.56, SD 1.04 years); 89% (16/18) of the participants were single; 61% (11/18) of the participants reported a medium level of health status, while 39% (7/18) indicated a good level of health status. Table 4 presents the demographic characteristics of the participants.

**Table 4:** Demographic information of interviewees (n=18).

Participant ID	Group	Gender	Age (years)	Marital status	Health status
1	IG1^a^	Female	19	Single	Medium
2	IG1	Male	20	Single	Medium
3	IG1	Male	19	Single	Medium
4	IG1	Male	19	Single	Medium
5	IG1	Female	20	Single	Good
6	IG1	Female	19	Single	Medium
7	IG2^b^	Male	18	Single	Good
8	IG2	Male	19	Single	Medium
9	IG2	Male	18	Single	Good
10	IG2	Female	20	Single	Medium
11	IG2	Female	19	Single	Medium
12	IG2	Female	21	In a relationship	Medium
13	CG^c^	Female	21	Single	Good
14	CG	Female	20	Single	Good
15	CG	Female	19	Single	Medium
16	CG	Male	20	Single	Good
17	CG	Female	22	Single	Good
18	CG	Male	19	In a relationship	Medium

^a^IG1: physical activity–first group.

^b^IG2: food-vegetable consumption–first group.

^c^CG: control group.

#### Major Themes

All participants were invited to talk about their experiences and participation in the web-based MHBC intervention program. Through thematic analysis, four major themes were identified: (1) PA and FVC behavior, (2) health-related outcomes, (3) correlates of health behavior change, and (4) contamination detection.

##### Theme 1: PA and FVC Behavior

This theme focused on how the students self-assessed their current status and changes in PA and FVC behavior over the previous 8 weeks. It contained three subthemes: (1) improving health behaviors, (2) no change in health behaviors, and (3) decrease in health behaviors.

###### Improving Health Behaviors

In total, 4 of the 12 (33%) students in the intervention groups reported improvement in their PA in the last 8 weeks (participants 1, 2, 4, and 6), while no student in the control group indicated an increase in this behavior. In addition, 2 students with improved PA stated that they understood the importance of performing sufficient PA through the web-based health program (participants 2 and 6). They noted some improvements, even after encountering obstacles at the start of implementation. For example:

I think the health program is quite helpful...I had not paid much attention to the health issue, especially since the last year of high school, you know, I was busy with my studies...But recently, thanks to the health learning sessions, I started to worry about my health status and I really improved this behavior these days, I feel that doing some physical activity makes all of my days...

Participant 6

For FVC, the feedback was more positive in the intervention groups, as 10 of the 12 (83%) students (except participants 1-4) described their improvement in the consumption of fruit and vegetables per day after receiving the health interventions. In comparison, only one student (participant 18) in the control group reported an increase in this behavior:

I eat more fruit and vegetables every day after participating in the health learning program...I pay more attention to this health issue now.

Participant 9

###### No Change in Health Behaviors

The results revealed that 5 of the 12 (41%) students (participants 3, 5, 7, 8, and 10) in the intervention groups reported maintaining their physical activity, while 2 of the 6 (33%) students in the control group reported no change in their weekly amount of PA (participants 17 and 18). The participants explained the following:

In the last two months, there has been no prominent change in this behavior...I maintain the same intensity and the same amount of weekly physical activity.

Participant 3

For FVC, two students (participants 1 and 4) in the intervention groups reported no change in their daily consumption of fruit and vegetables compared with four students in the control condition group (participants 13, 14, 16, and 17).

###### Decrease in Health Behaviors

Of the 12 students, 3 (25%) students in the intervention groups reported reducing the weekly amount of PA for diverse reasons, such as weather and study-related activities (participants 9, 11, and 12). Of the 6, 4 (66%) students in the control group also witnessed a decrease in PA in the last 8 weeks (participants 13-16). For FVC, only one student in the control group reported reducing her daily consumption of fruit and vegetables (participant 15): “I used to eat apples or grapes every day, but now I do not because of the freezing weather and other reasons.”

##### Theme 2: Health-Related Outcomes

This theme focused on how students self-assessed their physical and mental health outcomes. It contained three subthemes: *body weight*, *depression*, and *perceived quality of life*.

###### Body Weight

Of the 12 students, 4 (33%) in the intervention groups showed an increase in body weight (participants 2, 4, 9, and 11), and 3 of the 6 students (50%) in the control group described a similar trend (participants 13, 15, and 16). Two students in the intervention groups explained that their body weight increased because they were fitter and had more muscles these days (participants 2 and 4), but no further elaboration and explanation was obtained from the students in the control group. For example:

My body weight has increased a bit, but I think it is due to my muscles...there is no obvious change in my body fat.

Participant 2

###### Depression

Most of the students experienced no symptoms of depression in the previous 8 weeks (12/18, 66%). Although no student described the change in their level of depression, most students recognized the positive effects of PA and FVC in reducing depression. In particular, six students mentioned that PA can help fight depression (participants 2, 3, 5, 10, 17, and 18). Two students (participants 2 and 12) described the positive effect of FVC on reducing depression and felt that this influence was weaker than that of physical activity. For instance:

Doing exercise is useful for coping with depression and eating fruit and vegetables can put me in a good mood...But, I think that dietary behavior is not as effective as exercise in dealing with this problem...

Participant 12

###### Quality of Life

In total, 10 of the 18 (55%) students felt that their quality of life was good before participating in the health program. A total of 10 respondents (4 in the intervention groups and 6 in the control group) indicated no change in their quality of life. Two respondents indicated a decrease in this aspect (participants 9 and 11), and 6 respondents in the intervention groups (participants 2, 3, 5, 6, 8, and 12) felt that they were more energetic and their perceived well-being improved after participating in the web-based health program. In total, 9 of the 18 (50%) students recognized that consuming enough fruit and vegetables could help, while 6 students indicated the positive influence of regular PA on improving their perceived quality of life (Multimedia Appendix 8). For instance:

After participating in the health learning program, I exercised more, it brought me a good spiritual outlook...I felt that I slept better...Greasy food made me uncomfortable and fresh fruit and vegetables improved my well-being.

Participant 2

##### Theme 3: Correlates of Behavior Change

This theme reflected the students’ narratives of the correlates of health behavior change. It contained two subthemes: university policy for PA and barriers to PA and FVC.

###### University Policy for Physical Activity

The respondents mentioned that to encourage students to engage in PA, their university had a policy in place, named *Ham Run,* which was used as one of the assignments in the physical education class. The university declared that all undergraduates had to run 2000 m 28 times, accounting for 20% of the course credit. The students also indicated that they had to complete the *Ham Run* task before November, as the physical education course final usually takes place in mid-November:

The Ham Run task was okay for me, I completed it at the end of October...I feel that I was less active after completing this task.

Participant 5

###### Barriers to PA and FVC

*The s*tudents reported several barriers to their motivation and implementation of health behaviors. For physical activity, students mentioned the weather and facilities that inhibited their engagement in physical activity. For example:

The weather is freezing and the playground is quite far from my dormitory, so I rarely go outside to exercise...

Participant 9

Sometimes I want to exercise indoors, but the venues are always unavailable...it is really difficult.

Participant 3

For FVC, the barriers included the weather, the supply of university canteens, and financial issues. For instance, some students explained the following:

I do not want to eat vegetables because the weather is freezing and my teeth need hot food.

Participant 15

I cannot choose what I want to eat...it is decided by the university canteens...I hope they can improve their supply of vegetables.

Participant 4

The fruit sold nearby is quite expensive...I can only afford one serving of fruit per day...I do not want to ask my family for more money.

Participant 1

##### Theme 4: Contamination Detection

This theme consisted of three subthemes related to potential intervention contamination: *communication with classmates in the same physical education class*; *communication with classmates in a different physical education class*; and *communication with friends, roommates, and family*.

###### Communication With Classmates in the Same Physical Education Class

All students in the intervention and control groups indicated that they did not discuss the content of the health program with other classmates in the same PE class. The students provided additional information, emphasizing that there was a low possibility for students from the same department to enroll in the same PE class to discuss the content due to the university’s curriculum selection system. Some students explained the following:

I did not discuss the content with others, as I am not familiar with my classmates and we come from different departments...it is impossible for us (students in my department or roommates) to select the same PE class...

Participant 5

###### Communication With Students in a Different Physical Education Class

All students explained that they did not discuss the content with other students participating in the health program but were enrolled in a different PE class. For instance:

I do not know if the students enrolled in other PE classes were also invited to join the health program...I will not communicate with others, even if I find some acquaintances participating in this program.

Participant 7

###### Communication With Friends, Roommates, and Family

In total, 17 of the 18 students (94%) reported that they did not discuss the health program with their friends, roommates, and family. Only 1 student told her parents about her participation in the health program, but did not discuss the content of the intervention:

I told my mother that I participated in a health learning program and she encouraged me to adhere to it...but I did not give details.

Participant 2

## Discussion

### Principal Findings

For the quantitative part of the study, most of the research hypotheses were supported. For the qualitative analysis, 4 main themes with a couple of subthemes were identified through thematic analysis. The qualitative findings corresponded to the quantitative findings, providing an in-depth understanding of changes in PA and FVC behavior in Chinese college students.

### Intervention Effects on Behavioral Indicators

The principal expected intervention effects on the behavioral indicators of PA and FVC were identified in the quantitative study. From the findings of the RCT, compared with students in the placebo control condition, students in both intervention groups reported significant and favorable changes in the weekly amount of PA and daily consumption of fruit and vegetables, which supported hypothesis 1. The findings were more positive than those of our previous study, in which a significant treatment effect was only supported for FVC change in Chinese college students [24]. The treatment effects in this study were consistent with another study, which was conducted in Germany and the Netherlands, that aimed to improve the PA and FVC in adults who intended to reduce cardiovascular risk [54]. As the intervention materials were similar to those used in previous studies [54], the intervention effects on PA and FVC found in this study might be suitable for use in other Asian and European countries.

In terms of the differences in intervention effects on behavioral changes between the 2 delivery timings (PA-first vs FVC-first), hypothesis 3 was partially supported in the RCT. We found that there was no significant difference in PA between the 2 intervention groups at either time point, whereas the FVC-first group had significantly higher consumption of daily fruit and vegetables after 12 weeks than the PA-first group. Our finding is partially consistent with a previous study in middle-aged adults, which found that the PA-first group showed higher PA than the diet-first group (Cohen *d*=0.37, *P*<.001), while the diet-first group showed a higher FVC than the PA-first group (Cohen *d*=0.28, *P*<.001) after a 12-month intervention [23]. Our findings might be interpreted as compensatory effects [55]. It seems that FVC-first students in this study were more likely to consume more fruit and vegetables to compensate for the reduced PA compared with PA-first students. Nevertheless, this assumption has not been systematically examined in this study and deserves further investigation.

### Intervention Effects on Health-Related Outcomes

The findings of the RCT revealed that both intervention groups showed more changes in BMI compared with the control group, which partially supported hypothesis 2. As most of the participants were freshmen and sophomores and the data collection (ie, T3 and T4) was conducted near the beginning of the winter holidays, most of the participants increased their body weight because of the seasonal time that the study was conducted (special transition stage of life and seasonal variation) [56]. However, the students in the 2 intervention groups had significant reductions in the upward trend in BMI compared with the students in the control condition. This was consistent with findings from other MHBC studies, which suggested that combined PA and diet interventions had a more robust effect on weight management than interventions on either PA or diet alone in adults [57].

For depression, in the RCT, we did not find a significant effect, probably because of the floor effect [58], that is, the college students in this study reported a low incidence of depression (mean 0.92, SD 0.69; scale range from 0 to 3) at the beginning of the intervention. However, to prevent depression over the long term, more components that include stress management techniques and explicitly address mental health problems need to be developed and examined in future studies. The average score for quality of life was relatively high (3.15, SD 0.67; scale range from 1 to 5), which validated the baseline assessment of a high level of PA and a low level of depression among the participants. The ceiling effect might lead to the nonsignificance of the intervention on the perceived quality of life, coupled with the depression indicator, indicating the healthy mental states of these participants.

### Dropout Rate

It is not surprising that the dropout rates in this study were significantly lower than those in our previous study at both the postintervention test (26.3% vs 31.6%) and at the 1-month follow-up (37.9% vs 71.2%) [24]. As the psychosocial constructs and behavior change techniques were the same in our 2 studies, the higher retention rates might be attributable to the strategies applied in this study (eg, improvement of website design and technology and multiple reminders), which is consistent with other researchers’ suggestions [36]. However, the intervention and follow-up durations were comparatively short in this study, and the dropout issue still needs to be considered in future MHBC intervention programs.

### Qualitative Findings

The 4 themes provided a clear picture of the participants’ experiences and perceptions of participating in the web-based MHBC intervention program. The first theme reflected the perceived changes in PA and FVC of participants. Most of the students in the intervention groups indicated a favorable change in these 2 behaviors, especially for FVC (10/12, 83% indicated an increase). In contrast, most students in the control group showed a decrease in PA (4/6, 66%) and no improvement or decline in FVC (5/6, 83%). Theme 2 reflects participants’ perceptions of their change in health-related outcomes. Around half of the students mentioned an upward trend in body weight (7/18, 38%), most of whom indicated a decrease in PA (5/7, 71%). A total of 2 students who improved PA also showed a slight increase in their body weight and attributed it to muscle enhancement. No statistically significant effects on depression and quality of life were identified from the quantitative data, and we found that most students reflected a good knowledge of the benefits of adequate PA and FVC in depression (5/12, 41%) and quality of life (10/12, 83%) after receiving the web-based MHBC interventions. Despite no explicitly positive comments about the change in these 2 indicators, half of the students in the intervention groups described that they became more invigorated and had a better perceived quality of life after participating in the web-based health interventions.

From the qualitative study, we also found some additional information that underlined the crucial role of university policy in promoting physical activity and revealed prominent barriers to PA and FVC behavior. The students described the university’s relevant policy, which motivated their engagement in physical activity. To some extent, this can explain the situation in the previous RCT of participants reporting a relatively high amount of weekly physical activity. These results also echoed the suggestions from other studies, emphasizing the importance of including *sports time* in curricula and suggesting that supportive school policies should be considered when promoting the health of college students [5,59]. Furthermore, in the qualitative study, the students highlighted the extrinsic environmental factors obstructing the execution of PA and FVC, such as weather, facilities, and financial support. These barriers are consistent with findings from a previous qualitative study conducted in the United Kingdom, emphasizing the university environment and finance as barriers to students’ PA and dietary behavior [59].

Finally, theme 4 reflected the results of contamination detection, which provided qualitative evidence and explanation for a low risk of contamination in this study.

### Strength and Limitations

This study has considerable theoretical and practical implications for further web-based MHBC interventions. The use of the mixed methods approach increased the external validity of the quantitative data for factor-outcome relationship and thus was generalizable to a larger college student population and also ensured the strong internal validity of the in-depth descriptive qualitative data regarding complex context-specific issues and phenomena (eg, participating in a web-based MHBC intervention program) [60]. Despite the methodological merits and profound implications of the study findings, several limitations need to be addressed. First, all the research data were obtained via self-report measures or narratives, which may lead to recall bias and social desirability effects [61]. Furthermore, although a physical education course at a university in China provided a convenient setting for the RCT design, spillover and contamination could not be ignored [62]. Several strategies have been used to minimize this problem, and the follow-up interview did not identify any contamination; however, this issue still deserves further consideration (eg, adopting a cluster RCT). Furthermore, the intervention study was conducted during winter, where the effects might be confounded by seasonal factors. Further examination, including different seasonal contexts, is warranted. As with all qualitative research, the findings generated in this study could not be regarded as representative of all student samples who received a web-based MHBC intervention for PA and FVC, and caution is needed when generalizing from the interview sample to wider populations. In addition, given previous evidence and theoretical assumptions, this study focused only on a sequential delivery mode, comparing PA-first with FVC-first. However, to more comprehensively address the timing of MHBC intervention delivery, further studies should add a simultaneous design to compare the advantages of the 2 delivery timings and also consider participants’ preferences [63].

### Conclusions

Using a mixed methods approach, the study demonstrated the potential of a web-based and theory-based MHBC intervention for promoting both PA and FVC among Chinese college students. Moreover, the differences in intervention effects on changes in PA and FVC between the 2 delivery sequences were primarily identified in the quantitative study. In addition, the qualitative interviews provided an in-depth understanding of the quantitative findings and identified PA policy and external barriers as other determinants of change in PA and FVC. The overall findings provide new insights into MHBC research, providing theoretical and practical implications for future design and the application of web-based MHBC interventions.

## Appendix

Multimedia Appendix 1 Study process.

Multimedia Appendix 2 Intervention variables and content for the 2 intervention groups and the setting for a placebo control group.

Multimedia Appendix 3 Behavioral change techniques.

Multimedia Appendix 4 Interview guide.

Multimedia Appendix 5 Trustworthiness of the qualitative study.

Multimedia Appendix 6 Consolidated Criteria for Reporting Qualitative Studies (COREQ) checklist (32-item).

Multimedia Appendix 7 Sensitivity test using a per-protocol strategy.

Multimedia Appendix 8 A summary of the qualitative findings.

Multimedia Appendix 9 CONSORT-eHEALTH checklist (V 1.6.1).

## Abbreviations

FVC: fruit-vegetable consumption
HAPA: health action process approach
MHBC: multiple health behavior change
PA: physical activity
PE: physical education
RCT: randomized controlled trial
VAS: visual analogue scale
