# Supplementary figures and images for: The Effectiveness of Sequentially Delivered Web-Based Interventions on Promoting Physical Activity and Fruit-Vegetable Consumption Among Chinese College Students: Mixed Methods Study
Source: J Med Internet Res. 2022 Jan 26;24(1):e30566. doi: 10.2196/30566 (PMC8829698; doi:10.2196/30566)

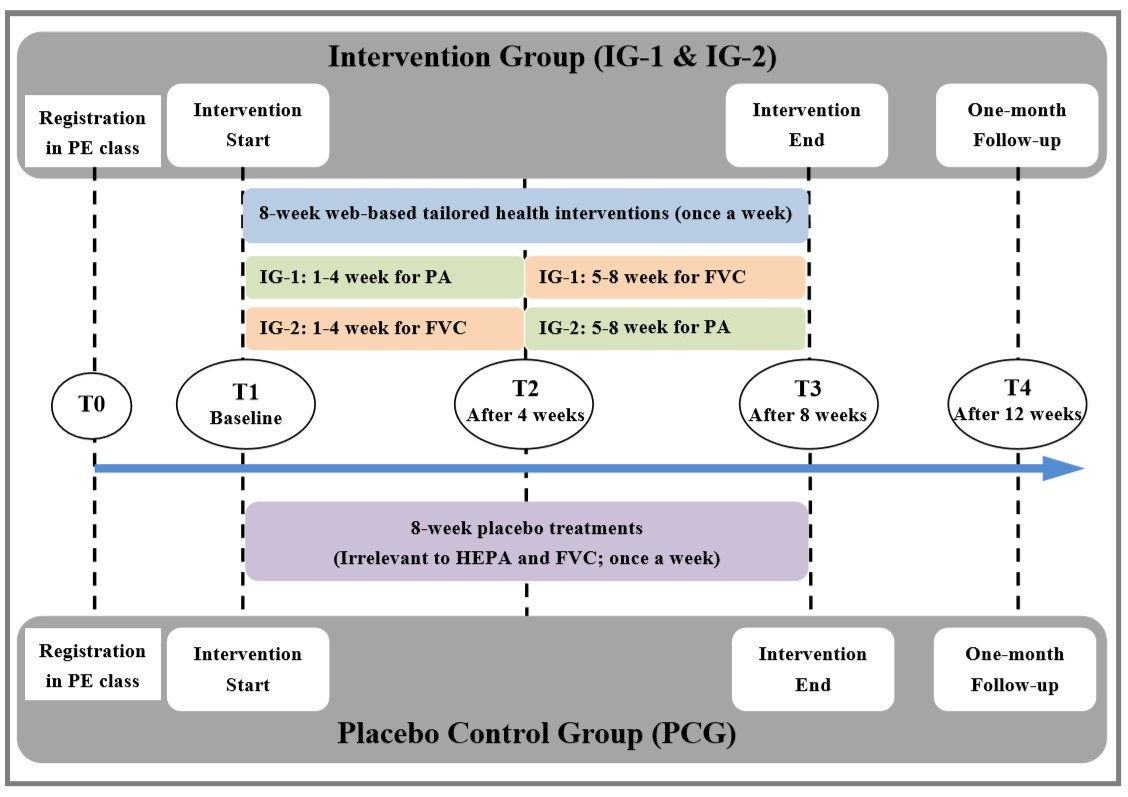

Supplement: Multimedia Appendix 1 [file jmir_v24i1e30566_app1.png]
