# Supplementary material for: The Effectiveness of Sequentially Delivered Web-Based Interventions on Promoting Physical Activity and Fruit-Vegetable Consumption Among Chinese College Students: Mixed Methods Study
Source: J Med Internet Res. 2022 Jan 26;24(1):e30566. doi: 10.2196/30566 (PMC8829698; doi:10.2196/30566)
Supplement: Multimedia Appendix 2 [file jmir_v24i1e30566_app2.docx]

| **IG-1 (1-4 week: PA; 5-8 week: FVC)** | **Intervention Variables** | **Physical activity (PA)** | | | | **Fruit and vegetable consumption (FVC)** | | | |
| --- | --- | --- | --- | --- | --- | --- | --- | --- | --- |
|  |  | Week 1 | Week 2 | Week 3 | Week 4 | Week 5 | Week 6 | Week 7 | Week 8 |
|  | Risk perception | ✓✓ |  |  |  | ✓✓ |  |  |  |
|  | Outcome expectancies | ✓✓ |  |  |  | ✓✓ |  |  |  |
|  | Self-efficacy | ✓✓ | ✓✓ | ✓✓ | ✓✓ | ✓✓ | ✓✓ | ✓✓ | ✓✓ |
|  | Goal setting | ✓✓ |  |  |  | ✓✓ |  |  |  |
|  | Action planning |  | ✓✓ | ✓✓ |  |  | ✓✓ | ✓✓ |  |
|  | Coping planning |  |  | ✓✓ | ✓✓ |  |  | ✓✓ | ✓✓ |
|  | Social support |  |  |  | ✓✓ |  |  |  | ✓✓ |
|  |  |  |  |  |  |  |  |  |  |
| **IG-2 (1-4 week: FVC; 5-8 week: PA)** | **Intervention Variables** | **Fruit and vegetable consumption (FVC)** | | | | **Physical activity (PA)** | | | |
|  |  | Week 1 | Week 2 | Week 3 | Week 4 | Week 5 | Week 6 | Week 7 | Week 8 |
|  | Risk perception | ✓✓ |  |  |  | ✓✓ |  |  |  |
|  | Outcome expectancies | ✓✓ |  |  |  | ✓✓ |  |  |  |
|  | Self-efficacy | ✓✓ | ✓✓ | ✓✓ | ✓✓ | ✓✓ | ✓✓ | ✓✓ | ✓✓ |
|  | Goal setting | ✓✓ |  |  |  | ✓✓ |  |  |  |
|  | Action planning |  | ✓✓ | ✓✓ |  |  | ✓✓ | ✓✓ |  |
|  | Coping planning |  |  | ✓✓ | ✓✓ |  |  | ✓✓ | ✓✓ |
|  | Social support |  |  |  | ✓✓ |  |  |  | ✓✓ |

Appendix 2: Intervention variables and content for two intervention groups and the setting for a placebo control group

| **IG-1: 1-4 week for PA; 5-8 week for FVC**  **(For IG-2, the content is same and only the delivery sequence is reversed)** | **Intervention Content** | Informing the risk of inactive behavior  Introducing and prompting the benefits of PA  Setting goals for PA and general health  Encouraging students to build confidence in PA | Learning how to make specific action plans for PA  Provide instruction on how, when and where to perform what type of PA  Taking examples of successful case about action plan execution  Prompting review of health goals  Prompting feedback on PA performance  Providing encouragement for performing PA | Reviewing the execution of action plans; adjusting action plans  Finding barriers or difficulties of plan execution; Setting coping plans  Taking examples of successful case about health behavior adherence to PA  Prompting review of health goals  Prompting feedback on PA performance  Providing encouragement for performing PA | Reviewing the execution of coping plans; adjusting coping plans  Prompting perceived social support on PA  Taking examples of successful case about behavior relapse prevention for PA  Prompting review of health goals  Prompting feedback on PA performance  Provide encouragement for performing PA | Informing the risk of unhealthy diet  Introducing and prompting the benefits of FVC  Setting goals for FVC and general health  Encouraging students to build confidence in FVC | Learning how to make specific action plans for FVC  Provide instruction on how, when and where to perform what type of FVC  Taking examples of successful case about action plan execution  Prompting review of health goals  Prompting feedback on FVC performance  Provide encouragement for performing FVC | Reviewing the execution of action plans; adjusting action plans  Finding barriers or difficulties of plan execution; Setting coping plans  Taking examples of successful case about health behavior adherence to FVC  Prompting review of health goals  Prompting feedback on FVC performance  Provide encouragement for performing FVC | Reviewing the execution of coping plans; adjusting coping plans  Prompting perceived social support on FVC  Taking examples of successful case about behavior relapse prevention for FVC  Prompting review of health goals  Prompting feedback on FVC performance  Provide encouragement for performing FVC |
| --- | --- | --- | --- | --- | --- | --- | --- | --- | --- |

| **Placebo control group** | **Placebo Treatments** | Leisure tourism | | Winter warmth | | Emotion regulation | | Music & Movie | |
| --- | --- | --- | --- | --- | --- | --- | --- | --- | --- |
|  |  | Introduction to five tourist attractions in Hong Kong | Introduction to five tourist attractions in Hong Kong | Five life tips for keeping warm in winter | Acupoint massage skill for keeping warm in winter | The usage of three essential oils for relaxation and emotion adjustment | The usage of three general psychological skills for regulating emotion | Introduction to three light music for relaxation | Introduction to three interesting movies for relaxation |
