# Supplementary material for: The Effectiveness of Sequentially Delivered Web-Based Interventions on Promoting Physical Activity and Fruit-Vegetable Consumption Among Chinese College Students: Mixed Methods Study
Source: J Med Internet Res. 2022 Jan 26;24(1):e30566. doi: 10.2196/30566 (PMC8829698; doi:10.2196/30566)
Supplement: Multimedia Appendix 3 [file jmir_v24i1e30566_app3.docx]

Appendix 3: BCTs

| **Behavior change techniques (BCTs)** | **Intervention session** |
| --- | --- |
| - Provide information about health consequences (e.g., risks of unhealthy behaviors in general with epidemiological data; benefits of health behaviors to the individual) (5.1) - Goal setting (e.g., PA and FVC behavior change; general health, weight management, energy balance) (1.1 & 1.3) | Week 1 & 5; |
| - Action planning (1.4) - Provide instruction on how to perform the behavior (4.1) | Week 2, 3, 6 &7; |
| - Problem solving (e.g., barrier identification/relapse prevention/coping planning) (1.2) - Discrepancy between current behavior and goal (1.6) | Week 3, 4, 7 & 8; |
| - Social support (unspecified, practical, emotional) (3.1, 3.2, 3.3) | Week 4 & 8; |
| - Prompt review of behavioral goals (1.5) - Prompt review of outcome goals (1.7) - Prompting feedback on performance (2.2) - Facilitate social comparison (e.g., take other people’s successful examples) (6.2) - Prompt social reward (e.g., encouraging praise) (10.4) - Prompt verbal persuasion about capability (e.g., tell the participants that they can successfully perform the wanted behavior) (15.1) | Week 2,3,4,6,7,&8; |
