# Supplementary material for: The Effectiveness of Sequentially Delivered Web-Based Interventions on Promoting Physical Activity and Fruit-Vegetable Consumption Among Chinese College Students: Mixed Methods Study
Source: J Med Internet Res. 2022 Jan 26;24(1):e30566. doi: 10.2196/30566 (PMC8829698; doi:10.2196/30566)
Supplement: Multimedia Appendix 4 [file jmir_v24i1e30566_app4.docx]

Appendix 4: Interview Outline (used by interviewer)

| **Opening** | Build Rapport; Purpose of the interview; Ethical Issues; Informed Consent; Timeline | 1. Thank you for coming and participating in this interview.  2. I would like to ask you some questions about your feelings as a user of our web platform and your experience of participating in our web-based health program.  3. I would like to record the interview as this helps us capture exactly what you say, but all private information will remain confidential. The interview data will only be used for academic research purposes and destroyed after 12 months.  4. Sign the informed consent form  5. This interview will last approximately 30 minutes, the monetary incentive (interviewee fee, RMB40) will be provided immediately after completing the interview. Feel free to ask questions at any stage during the interview.  6. Are you available to answer some questions at this stage? (Do you have any questions?) |
| --- | --- | --- |
| **Transition** | **A-Completers in IGs:** Thank you for completing the 8-week web-based health intervention and the 2-time survey. Let’s think about the web-based health learning content you received in the past eight weeks.  **B-Completers in CG:** Thank you for reading the 8-week recommended life tips and for completing the 2-time surveys. Let’s start with some questions about your experience with PA and FVC in the past eight weeks. | |
| **Topic** | Students’ experience of participating in the web-based MHBC intervention program | What is your experience of participating in the web-based health program (two intervention groups and one placebo-control group)?  1. Direct the subjects to talk about their current status and changes in PA and FVC by asking the following questions:  - What is your current PA situation, such as what type of PA do you engage in the most, with what intensity and frequency per week? Do you achieve the recommendation to perform at least 150 accumulated moderate PA per week? How long have you maintained this behavior?  - What is your current FVC situation, such as what type of fruit and vegetables do you consume the most, what portions and in what ways (e.g., raw fruit and vegetables, cooked or steamed vegetables or fruit-vegetable juice) per day? Do you achieve the recommendation to consume at least five servings of fruit and vegetables every day? How long have you maintained this behavior?  - With the web-based health program, has there been any change in your PA and FVC behaviors? (If so, can you explain in more detail? If not, tell me why) |
|  |  | 2. Direct the subjects to talk about their current status and changes in health outcomes by asking the following questions:  - How is your current body weight? Has your body weight changed in the past eight weeks? (If so, what caused this change?)  - Did you feel depressed last week? Do you feel hopeful about the future? Do you have trouble focusing on what you are doing? (If so, how often do you have these feelings?) Has there been any change in your level of depression after participating in the web-based health program? (If so, can you explain in more detail? If not, tell me why.)  - How would you rate your quality of life? Do you have enough energy for everyday life? How satisfied are you with your sleep and with your ability to study? Has there been any change in your quality of life after participating in the web-based health program? (If so, can you explain in more detail? If not, tell me why.)  3. To identify contamination between intervention and control groups:  - Where did you complete the questionnaire survey and health program (e.g., in the dormitory or at home)?  - Did you complete it independently? Did you discuss the content of the program with your classmates in the same PE class?  - Did you discuss the content of the program with your classmates who participated in this program but were enrolled in a different PE class?  - Did you discuss the content of the program with other friends, roommates or family members? |
| **Closing** | Is there anything else you would like to add to the question? I appreciate the time you tool for this interview. Here is your interviewee fee.  Thank you! | |
