# Supplementary material for: The Effectiveness of Sequentially Delivered Web-Based Interventions on Promoting Physical Activity and Fruit-Vegetable Consumption Among Chinese College Students: Mixed Methods Study
Source: J Med Internet Res. 2022 Jan 26;24(1):e30566. doi: 10.2196/30566 (PMC8829698; doi:10.2196/30566)
Supplement: Multimedia Appendix 5 [file jmir_v24i1e30566_app5.docx]

**Trustworthiness of the findings**

Efforts were made to ensure the credibility and trustworthiness of the qualitative results, according to the principles of : sensitivity, commitment, rigor, transparency, coherence, impact and importance (Yardley, 2017).

Sensitivity: both inductive and deductive processes were used for data analysis, in which the codes and themes were extracted and defined based on a careful examination of the meanings generated by the participants.

Commitment and rigor: the researchers showed an in-depth engagement with the topics, conducted a thorough data collection, had expertise and experience in the methods used, and undertook a detailed and in-depth analysis of the qualitative data.

Transparency and coherence: as triangulation approaches for data validation, peer review and comparison with previous quantitative results enabled transparency and coherence, confirming the results and offering different perspectives.

Impact and importance: the results of this study provided additional information to the previous quantitative study and contributed to the future design and refinement of web-based MHBC interventions for college students.
