# Supplementary material for: The Effectiveness of Sequentially Delivered Web-Based Interventions on Promoting Physical Activity and Fruit-Vegetable Consumption Among Chinese College Students: Mixed Methods Study
Source: J Med Internet Res. 2022 Jan 26;24(1):e30566. doi: 10.2196/30566 (PMC8829698; doi:10.2196/30566)
Supplement: Multimedia Appendix 6 [file jmir_v24i1e30566_app6.docx]

*32-item Consolidated criteria for reporting qualitative studies (COREQ)*

| **No. Item** | **Guide questions/description** | **Reported on Manuscript** |
| --- | --- | --- |
| **Domain 1: Research team and reﬂexivity** |  |  |
| *Personal Characteristics* |  |  |
| 1. Interviewer/facilitator | Which author/s conducted the interview or focus group? | Prior to the formal interview, the interviewer (WL) has conducted pilot interviews with three college students in order to familiarize with the interview guide and procedure. |
| 2. Credentials | What were the researcher’s credentials? E.g. PhD, MD | The moderator and interviewer held PhD candidature. |
| 3. Occupation | What was their occupation at the time of the study? | The interviewer and the moderator were PhD students. |
| 4. Gender | Was the researcher male or female? | The moderator and interviewer was male. |
| 5. Experience and training | What experience or training did the researcher have? | Academic training and past experience in qualitative research methods, as well as a series of pilot interviews |
| ***Relationship with participants*** |  |  |
| 6. Relationship established | Was a relationship established prior to study commencement? | No prior relationship. |
| 7. Participant knowledge of the interviewer | What did the participants know about the researcher? E.g., personal goals, reasons for doing the research | No prior knowledge of the interviewer. |
| 8. Interviewer characteristics | What characteristics were reported about the interviewer/facilitator? E.g., Bias, assumptions, reasons and interests in the research topic | All the authors had a neutral attitude towards health program, and strived to remain neutral in the conversations with participants. |
| **Domain 2: study design** |  |  |
| *Theoretical framework* |  |  |
| 9. Methodological orientation and Theory | What methodological orientation was stated to underpin the study? e.g. grounded theory, discourse analysis, ethnography, phenomenology, content analysis | Thematic Analysis. |
| *Participant selection* |  |  |
| 10. Sampling | How were participants selected? e.g. purposive, convenience, consecutive, snowball | Purposive sampling. |
| 11. Method of approach | How were participants approached? e.g. face-to-face, telephone, mail, email | Participants were recruited from previous intervention study via email and telephone message. |
| 12. Sample size | How many participants were in the study? | 18 participants. |
| 13. Non-participation | How many people refused to participate or dropped out? Reasons? | For the main interviews, all eligible participants were firstly informed about the purpose of the interview with a participant information form and an informed consent form. |
| *Setting* |  |  |
| 14. Setting of data collection | Where was the data collected? e.g. home, clinic, workplace | The venues of interviews were arranged in quiet places within or nearby university (e.g., discussion room of library, cafe) according to the interviewees’ convenience and preference. |
| 15. Presence of non-participants | Was anyone else present besides the participants and researchers? | Only the researchers were present during data collection with the participants. |
| 16. Description of sample | What are the important characteristics of the sample? e.g. demographic data, date | The details about study sample are provided in Table 6 |
| *Data collection* |  |  |
| 17. Interview guide | Were questions, prompts, guides provided by the authors? Was it pilot tested? | An interview guide was developed by the research team jointly, including the questions, prompts and guides, based on the suggestions from Bryman (2016) and Flick (2009), |
| 18. Repeat interviews | Were repeat interviews carried out? If yes, how many? | No. |
| 19. Audio/visual recording | Did the research use audio or visual recording to collect the data? | Each interview was audio-recorded and lasted for around 30 minutes. |
| 20. Field notes | Were ﬁeld notes made during and/or after the interview or focus group? | No. |
| 21. Duration | What was the duration of the inter views or focus group? | Each interview was audio-recorded and lasted for around 30 minutes. |
| 22. Data saturation | Was data saturation discussed? | In order to achieve the theoretical saturation, based on the “rule of thumb” and “calculating the mean of selected qualitative studies”, the suggested number of participants for the interview studies is around 12-15 in homogeneous groups |
| 23. Transcripts returned | Were transcripts returned to participants for comment and/or correction? | No. |
| **Domain 3: analysis and ﬁndings** |  |  |
| *Data analysis* |  |  |
| 24. Number of data coders | How many data coders coded the data? | Two research members conducted the analysis procedure independently. |
| 25. Description of the coding tree | Did authors provide a description of the coding tree? | No. |
| 26. Derivation of themes | Were themes identiﬁed in advance or derived from the data? | Themes were derived from the data. |
| 27. Software | What software, if applicable, was used to manage the data? | QSR NVivo 11. |
| 28. Participant checking | Did participants provide feedback on the ﬁndings? | No. |
| *Reporting* |  |  |
| 29. Quotations presented | Were participant quotations presented to illustrate the themes/ﬁndings? Was each quotation identiﬁed? e.g. participant number | Yes. |
| 30. Data and ﬁndings consistent | Was there consistency between the data presented and the ﬁndings? | Yes. |
| 31. Clarity of major themes | Were major themes clearly presented in the ﬁndings? | Yes. |
| 32. Clarity of minor themes | Is there a description of diverse cases or discussion of minor themes? | Yes. |

Reference: Tong A, Sainsbury P, Craig J. Consolidated criteria for reporting qualitative research (COREQ): a 32-item checklist for interviews and focus groups. *International Journal for Quality in Health Care*. 2007. Volume 19, Number 6: pp. 349 – 357.
