# Supplementary material for: The Effectiveness of Sequentially Delivered Web-Based Interventions on Promoting Physical Activity and Fruit-Vegetable Consumption Among Chinese College Students: Mixed Methods Study
Source: J Med Internet Res. 2022 Jan 26;24(1):e30566. doi: 10.2196/30566 (PMC8829698; doi:10.2196/30566)
Supplement: Multimedia Appendix 7 [file jmir_v24i1e30566_app7.docx]

*Sensitivity analyses for the intervention effects with per-protocol approach (n = 343)*

| **Variable** | **Time × Group** | | **Time** | | **Group** | |
| --- | --- | --- | --- | --- | --- | --- |
|  | **Estimate** | ***P*** | **Estimate** | ***P*** | **Estimate** | ***P*** |
| PA | F = 2.25 | **.038** | F = 2.98 | .031 | F = 1.10 | .34 |
| FVC | F = 12.35 | **< .001** | F = 41.26 | < .001 | F = 16.96 | < .001 |
| BMI | F = 2.78 | **.012** | F = 16.34 | < .001 | F = 0.33 | .72 |
| Depression | F = 0.74 | .62 | F = 8.15 | < .001 | F = 3.24 | .041 |
| Quality of life | F = 2.38 | **.029** | F = 5.57 | .001 | F = 2.86 | .059 |

*Note.* PA = weekly amount of physical activity (MET-minute/week); FVC = daily servings of fruit-vegetable consumption (portion/day); BMI = body mass index (kg/m^2^).
