# Supplementary material for: The Effectiveness of Sequentially Delivered Web-Based Interventions on Promoting Physical Activity and Fruit-Vegetable Consumption Among Chinese College Students: Mixed Methods Study
Source: J Med Internet Res. 2022 Jan 26;24(1):e30566. doi: 10.2196/30566 (PMC8829698; doi:10.2196/30566)
Supplement: Multimedia Appendix 8 [file jmir_v24i1e30566_app8.docx]

Appendix 8: A summary of the qualitative findings.

| **Subject** | **PA** | **FVC** | **Body weight** | **Depression** | **Quality of life** | **University policy for PA** | **Barriers to PA and FVC** | **Contamination** |
| --- | --- | --- | --- | --- | --- | --- | --- | --- |
| #1 | + | 0 | 0 | 0 | 0 ΔPA influence | Δ | ΔFVC barriers | N |
| #2 | + | + | + Δreason | 0 ΔPA influence ΔFVC influence | + ΔPA influence ΔFVC influence | Δ | NA | N |
| #3 | 0 | + | 0 | 0 ΔPA influence | + ΔFVC influence | NA | ΔPA barriers | N |
| #4 | + | 0 | + Δreason | 0 | 0 ΔPA influence | NA | ΔFVC barriers | N |
| #5 | 0 | + | 0 | 0 ΔPA influence | + ΔFVC influence | Δ | ΔPA barriers | N |
| #6 | + | + | 0 | 0 | + ΔPA influence ΔFVC influence | Δ | NA | N |
| #7 | 0 | + | 0 | 0 | 0 ΔFVC influence | NA | NA | N |
| #8 | 0 | + | 0 | 0 | + ΔPA influence ΔFVC influence | NA | NA | N |
| #9 | - | + | + | 0 | - | NA | ΔPA barriers | N |
| #10 | 0 | + | 0 | 0 ΔPA influence | 0 ΔFVC influence | Δ | NA | N |
| #11 | - | + | + | 0 | - | NA | NA | N |
| #12 | - | + | 0 | 0 ΔFVC influence | + ΔFVC influence | Δ | ΔPA barriers | N |
| #13 | - | 0 | + | 0 | 0 | NA | ΔFVC barriers | N |
| #14 | - | 0 | 0 | 0 | 0 | Δ | NA | N |
| #15 | - | - | + | 0 | 0 | NA | ΔFVC barriers | N |
| #16 | - | 0 | + | 0 | 0 | NA | ΔPA barriers | N |
| #17 | 0 | 0 | 0 | 0 ΔPA influence | 0 ΔPA influence | Δ | NA | N |
| #18 | 0 | + | 0 | 0 ΔPA influence | 0 ΔFVC influence | Δ | NA | N |

Note. +: increase; - : decrease; 0: no change; Δ: provide with narrative elaboration; NA= not applicable (not mentioned); N = no.
